# A *GATA3* gene mutation that causes incorrect splicing and HDR syndrome: a case study and literature review

**DOI:** 10.3389/fgene.2023.1254556

**Published:** 2023-08-25

**Authors:** Yilun Tao, Lin Yang, Dong Han, Chen Zhao, Wenxia Song, Haiwei Wang, Xiaoze Li, Lihong Wang

**Affiliations:** ^1^ Medical Genetic Center, Changzhi Maternal and Child Health Care Hospital, Changzhi, Shanxi, China; ^2^ Department of Pediatrics, Changzhi Maternal and Child Health Care Hospital, Changzhi, Shanxi, China; ^3^ Obstetrics Department, Changzhi Maternal and Child Health Care Hospital, Changzhi, Shanxi, China; ^4^ Science and Education Division, Changzhi Maternal and Child Health Care Hospital, Changzhi, Shanxi, China

**Keywords:** GATA3 gene, HDR syndrome, next-generation sequencing, splice site variant, review

## Abstract

Hypoparathyroidism, deafness, and renal dysplasia (HDR) syndrome is an infrequent autosomal dominant genetic disorder caused by haploinsufficiency of the GATA binding protein 3 (*GATA3*) gene. In this report, we present a case study of a 6-year-old female patient manifesting seizures, tetany, hypoparathyroidism, and sensorineural hearing loss. A heterozygous variant, c.1050 + 2T>C, in the *GATA3* gene was discovered by genetic testing. Moreover, a minigene splicing experiment revealed that the aforementioned variation causes incorrect splicing and premature cessation of protein synthesis. The clinical profile of the patient closely resembles the well-known phenomenology of HDR syndrome, supporting the association between the condition and the *GATA3* variant. The challenges in early diagnosis highlight the importance of employing next-generation sequencing for timely detection of rare diseases. Additionally, this research contributes to a deeper understanding of the genotype-phenotype correlations in HDR syndrome, underscoring the critical need for improved diagnostic and therapeutic strategies.

## Introduction

Hypoparathyroidism, sensorineural deafness, and renal dysplasia (HDR, MIM 146255) syndrome is a rare autosomal dominant disorder that is characterized by the triad of hypoparathyroidism, sensorineural deafness, and renal dysplasia, accompanied by additional variable features, including cognitive disability, polycystic ovaries, congenital heart disease, and retinitis pigmentosa (Barakat et al., 2018). Although the precise prevalence of HDR syndrome remains uncertain, it is widely acknowledged as an exceedingly rare condition.

Thus far, only 191 cases of HDR syndrome have been documented in the scientific literature. The initial documentation of HDR syndrome dates back to 1977, when cases were identified in an American family (Hasegawa et al., 1997). Until 2000, the genetic basis of this disorder was linked to mutations in the *GATA3* gene (MIM 131320), which is located on chromosome 10p14 and encodes a dual zinc-finger transcription factor. The C-terminal zinc finger (ZnF2, residues 317-341), encoded by exon 5, plays a critical role in DNA binding. And the N-terminal zinc finger (ZnF1, residues 263-287), encoded by exon 4, helps to interact with other zinc finger proteins of different types and stabilizes DNA binding (Nesbit et al., 2004; Zahirieh et al., 2005; Lentjes et al., 2016). Various types of *GATA3* mutations have been identified, including intragenic deletions, frameshift, nonsense, acceptor splice site, and missense mutations (Nesbit et al., 2004). Although certain splice site mutations have been reported, the specific mRNA changes they induce have not been clearly defined (Chiu et al., 2006; Nakamura et al., 2011; Belge et al., 2017; Matsuo et al., 2017; Van Heurck et al., 2021).

In this study, we present a splice site variant, c.1050 + 2T>C, in the *GATA3* gene. Our investigation focused on elucidating the underlying molecular mechanisms responsible for the abnormal mRNA splicing caused by this variant, leading to an aberrant splicing pattern and resulting in a truncating mutation at the cDNA level (c.1050_1051ins80bp, p.Ile351Alafs*14). Furthermore, we conducted a comprehensive review, encompassing the clinical and genetic characteristics of previously reported cases of HDR syndrome. Through this extensive analysis, we aimed to provide valuable insights into the intricate relationship between genotype and phenotype in HDR syndrome, thereby advancing our understanding of this unique disorder.

## Materials and methods

### Case report

The proband, a 6-year-old girl, was admitted to the emergency unit due to episodes of seizures. These episodes had been occurring since the age of 7 months, which were suspected to be triggered by meningitis resulting from a respiratory tract infection. A computed tomography (CT) scan of the brain revealed an enlarged extracerebral interspace. She is the second-born child of healthy and unrelated Chinese parents with no known history of genetic diseases or maternal exposure to teratogenic pathogens or drugs during pregnancy. The proband was born at 38 weeks gestation weighing 3,000 g (50th percentile) and measuring 50 cm (75th percentile). The newborn hearing screening test failed at the age of 10 days. She first showed signs of speech at the age of 9 months, using words like “baba” and “mama”. At the age of 1 year and 5 months, she was diagnosed with bilateral sensorineural hearing loss, with a hearing loss of 60 dB in the right ear and 40 dB in the left ear. She started wearing a hearing aid and was able to speak three-word utterances at the age of 2. Her cognitive development was within the normal range by the time she was two and a half, and she could articulate coherent sentences proficiently. However, at the age of four, she experienced a recurrence of seizures accompanied by frequent falls while walking, recurrent muscle cramps, brief respiration pauses lasting a few seconds, and bouts of unconsciousness. A baseline electroencephalogram (EEG) showed normal results.

The proband had pyrexia and a cough after being admitted to the emergency department, but she was still conscious and his vital signs were stable. Despite normal muscular tonicity, there was facial myoclonus, lateralized ocular and oral asymmetry, muscle asthenia, and suboptimal motor coordination. The presence of influenza A viral infection in the patient was confirmed by viral culture and RT-PCR assays targeting respiratory pathogens. Routine hematological analysis, including a complete blood count and assessment of inflammatory markers, indicating mild hepatocellular derangement and myocardiocyte injury, potentially attributed to the influence of influenza A viral infection. The proband consistently exhibited low calcium levels accompanied by high phosphate levels, mild magnesium deficiency, and suppressed parathyroid hormone levels. Renal function and urine analysis, as well as arterial blood gas analysis, demonstrated normal results. Abdominal ultrasound showed no morphological abnormalities in the kidneys. Detailed laboratory assessments are provided in Table 1.

**TABLE 1:** Results of laboratory investigations in the proband.

Test items	7 months	6 years	After treatment	Reference range
Serum corrected calcium (mmol/L)	1.82	1.1	2.04	2.10–2.80
Serum phosphate (mmol/L)	2.03	3.14	2.7	1.05–2.15
Serum magnesium (mmol/L)	1.13	0.66	0.81	0.84–1.05
Serum potassium (mmol/L)	5.09	4.36	4.29	3.5–5.3
Serum sodium (mmol/L)	137.6	143	138	137–147
Serum chlorine (mmol/L)	102.6	107	104	1.03–1.35
Parathyroid hormone (PTH) (pg/mL)	N/A	1.1	1.31	1.17–8.59
Total 25-hydroxy vitamin D (ng/mL)	N/A	6.15	10.88	20–50
Serum creatinine (μmol/L)	32.2	28.53	31.6	27–62
Blood urea (mmol/L)	2.83	3.83	6.54	1.7–8.3
uric acid (μmol/L)	309.7	152.5	309.9	178–357
ALT (U/L)	210.1	60.5	10.6	3–40
AST (U/L)	164.5	67.3	24.2	14–44
ALT/AST	1.28	1.11	2.28	—
ALP (U/L)	193.1	142.3	192.1	143–406
CK (U/L)	62.5	1014.1	221.8	24–700
CK-MB (U/L)	39.6	33.7	22.1	0–24

N/A, not applicable.

Following admission, the patient received a daily intravenous infusion of 10 mg of 10% calcium gluconate for a 10-min period. The therapeutic regimen was amended to include oral calcium supplements at a prescribed dosage of 450 mg per day, in conjunction with 1,25-hydroxyvitamin D at a recommended dosage of 90 IU per day, starting on the second day of hospitalization. The instituted treatment yielded notable amelioration of the child’s clinical manifestations, accompanied by a marked elevation in the serum total calcium level, reaching 2.7 mmol/L. Furthermore, intravenous administration of sodium glucuronate at a daily dose of 100 mg and fructose diphosphate at a daily dose of 3 g was initiated and continued for a duration of 5 days. The patient also received oral administration of 45 mg of oseltamivir phosphate, twice daily, to effectively combat viral infection.

### Genetic analysis

Targeted next-generation sequencing was carried out by MyGenostics (MyGenostics Inc., Beijing, China). A peripheral blood sample was collected from the patient, and DNA extraction was performed utilizing the QIAamp DNA Mini Kit (Qiagen, China). The DNA library preparation adhered to Illumina protocols. For capturing the enriched libraries, a genetic diagnostic panel for hearing loss, encompassing 406 genes including the *GATA3* gene, was utilized. The sequencing itself was conducted on the Illumina HiSeq X Ten platform, generating 150 bp paired-end reads. Subsequently, the obtained reads were aligned to the UCSC hg19 human reference genome utilizing the Burrows-Wheeler Alignment tool. Classification of variants (pathogenic, likely pathogenic, VUS and likely benign and benign) has been done according to the variant interpretation guidelines of American College of Medical Genetics and Genomics (Richards et al., 2015). To confirm the candidate variable sites, Sanger sequencing was conducted on the patient, his brother, and his parents.

### Plasmid construction and transfection

For the minigene splicing assay, we employed a splicing reporter plasmid known as pcMINI-C (Bioeagle, China). To construct the pcMINI-C-*GATA3*-wt (wild-type) and pcMINI-C-*GATA3*-mut (mutant) plasmids, we cloned the entire exon 5 along with the flanking intron 4 and intron 5, as well as exon 6 sequences of both the wild-type and mutant *GATA3* into the pcMINI-C vector. The amplification of *GATA3* was carried out using specific primers, the nucleotide sequences of which can be found in Supplementary Table S1. Subsequently, the HEK293T and HeLa cells were transfected with pcMINI-C-*GATA3*-wt and pcMINI-C-*GATA3*-mut, respectively, using Metafectene (Biontex, Germany) following the instructions provided by the manufacturer.

### Minigene splicing assay

After 48 h of transfection, total RNA was extracted from both HEK293T and HeLa cells using TRIzol reagent (Takara, Japan). To eliminate any remaining DNA, the extracted RNA was treated with DNase I (Thermo Scientific, United States). Subsequently, the RNA was reverse transcribed into cDNA using the ImProm-II™ Reverse Transcription System, following the manufacturer’s instructions (Promega, United States). To investigate the alterations in splicing, specific cDNA fragments corresponding to the minigene constructs were amplified using primers designed for the respective plasmids. The amplified products were then separated through 12% agarose gel electrophoresis (AGE). In order to further characterize the splicing pattern, the PCR products were subjected to Sanger sequencing, which facilitated the identification of any splicing changes or abnormalities.

## Results

### Genetic testing

**FIGURE 1:**
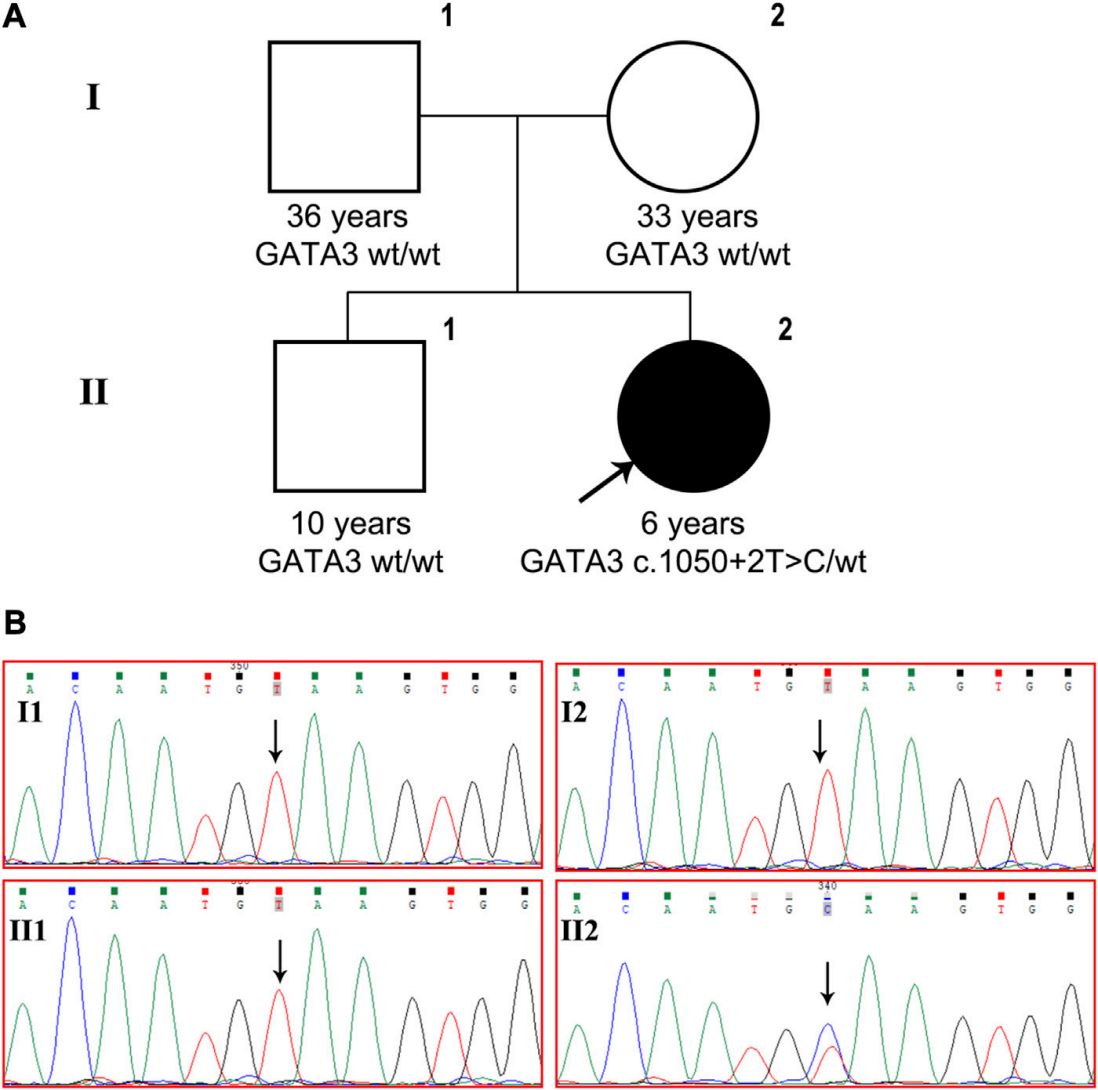
*GATA3* c.1050 + 2T>C was detected in a Chinese patient with HDR syndrome. **(A)** Pedigree of the family indicating the proband (black arrow, II-2). **(B)** *GATA3* gene sequencing of the patient, his brother and parents. The patient carried heterozygous *GATA3* mutation c.1050 + 2T>C, while the variant was not detected in her older brother or her parents.

A *de novo* heterozygous variant, c.1050 + 2T>C, was detected in intron 5 of the *GATA3* gene (NM_001002295) and validated using Sanger sequencing (Figure 1). This variant has not been previously reported in the literature and is not present in the general population or the HGMD database. In silico analysis using SPliceAI and NNSPLICE tools suggests that the variant may have an impact on pre-mRNA splicing, with scores of 0.96 and 0.13, respectively. The CADD value obtained through VarSome, which is an indicator of pathogenicity, was 33 for the variant. Based on the ACMG classification, the variant is predicted to be “pathogenic” (PVS1+PM2_Supporting + PP3+PP4). Based on molecular genetics studies and clinical evidence, the proband’s diagnosis of HDR syndrome was confirmed.

### Minigene splicing assay

**FIGURE 2:**
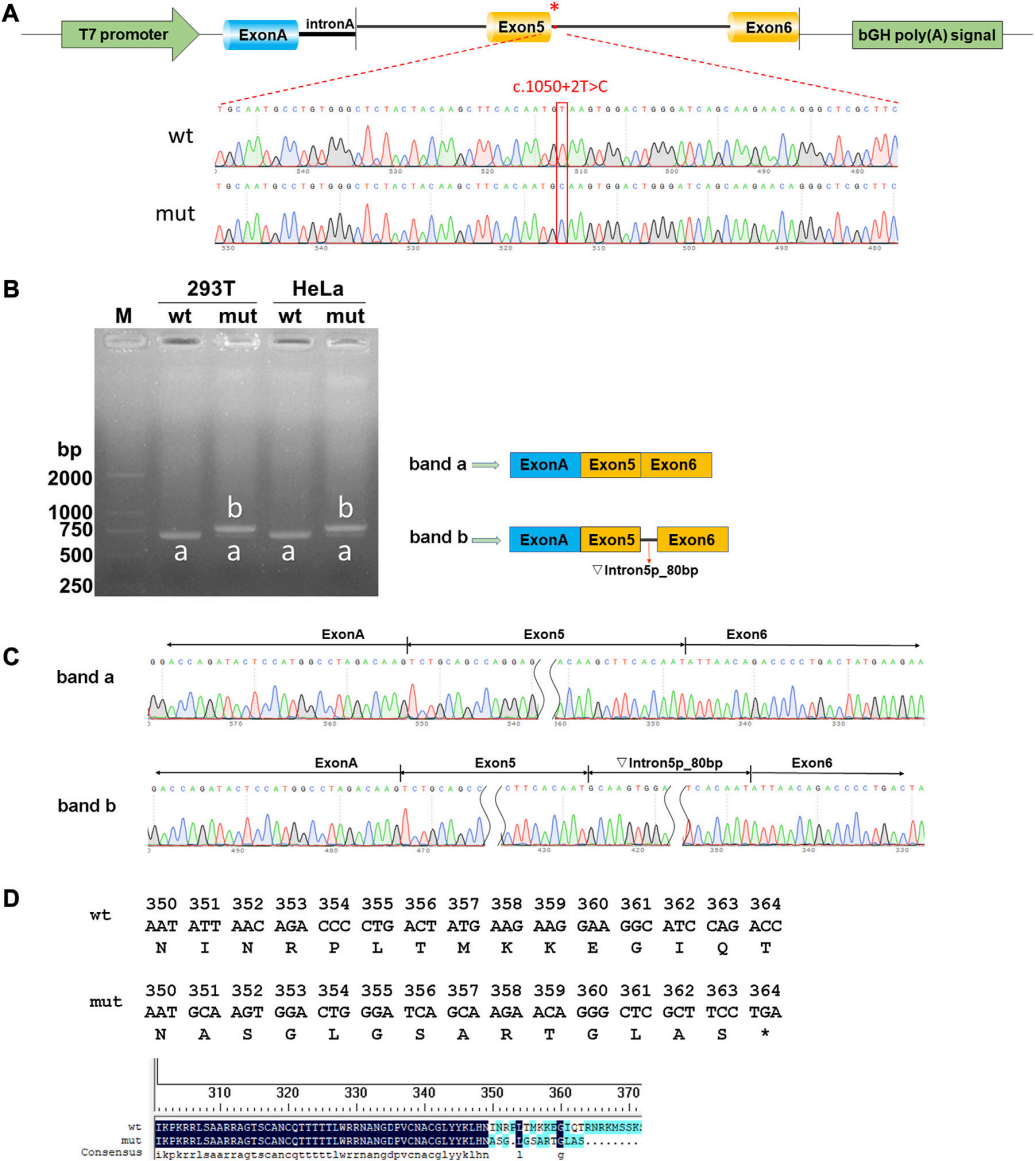
Results of the minigene splicing assay for the GATA3 c.1050 + 2T>C variant. **(A)** Illustration of vector construction: the pcMINI-C vector, containing the intron A and exon A sequences, was utilized for the cloning of the complete exon 5, along with the surrounding intron 4 and intron 5, as well as exon 6 regions of both the wild-type (wt) and mutant (mut) *GATA3*. **(B)** Analysis of expressed mRNA through agarose gel electrophoresis. The findings revealed distinct bands. Band a, corresponding to the wild-type minigenes, exhibited an expected size of approximately 603 bp in HEK293T or MCF-7 cells. Conversely, band b, representing the mutant minigenes, displayed a significantly larger size of 683 bp in both cell lines. **(C)** Analysis of mRNA using RT-PCR and direct sequencing. Sanger sequencing unveiled that band a manifested a normal splicing pattern [ExonA(192 bp)-Exon5(126 bp)-Exon6(285 bp)]. In contrast, band b exhibited an aberrant splicing pattern, implying that the *GATA3* c.1050 + 2T>C variant potentially disrupted the regular splicing pattern of exon 5. Consequently, an 80 bp segment of intron 5 was retained, leading to the generation of an anomalous GATA3 mRNA transcript: [ExonA(192 bp)-Exon5(126 bp)-∇intron5(80 bp)-Exon6(285 bp)]. **(D)** The amino acid sequences of wild-type and mutated proteins.

Both wild-type and mutant *GATA3* exons 5 and 6, as well as their adjacent introns 4 and 5 were successfully inserted into the pcMINI-C reporter vector (Figure 2A). The resulting constructs were then transfected into HEK293T and HeLa cells, respectively. Subsequent analysis of the resulting minigene-spliced RNA via RT-PCR revealed distinct band patterns. In particular, the wild-type construct produced the expected band (band a), while the mutant construct exhibited a larger band (band b) (Figure 2B). Sanger sequencing was performed to confirm the splicing patterns, indicating that band a represented a normal splicing pattern comprising ExonA (192 bp), Exon5 (126 bp), and Exon6 (285 bp). In contrast, band b showed the retention of a partial intron 5 (80 bp), resulting in an altered splicing pattern [ExonA(192 bp)-Exon5(126 bp)-∇intron5(80 bp)-Exon6(285 bp)] (Figure 2C). The impact of the variant on *GATA3* mRNA splicing was consistent across different cell types, including HEK293T and HeLa cells. The results demonstrated that the *GATA3* c.1050 + 2T>C variant elicited an aberrant splicing pattern with intron retention *in vitro*. Specifically, a partial intron 5 (80 bp) was retained between exons 5 and 6 (c.1050_1051ins80bp). Because of the intron retention, isoleucine was replaced with alanine at the 351st amino acid position in GATA3, leading in the formation of a premature termination codon (PTC). As a result, the *GATA3* c.1050 + 2T>C variant hindered the normal expression of *GATA3*, leading to the production of a truncated GATA3 protein (p.Ile351Alafs*14, Figure 2D).

## Discussion

This study presents a compelling case of a patient diagnosed with hypoparathyroidism, sensorineural hearing loss, recurring seizures, basal ganglia calcification, muscle cramps, and apnea. Notably, c.1050 + 2T>C, a heterozygous variant in the *GATA*3 gene that had never been described in the scientific literature and was absent from the general population, was discovered. Notable findings from bioinformatics study point to a potential role for this variation in controlling pre-mRNA splicing. Furthermore, compelling evidence has demonstrated that this variation can effectively induce premature termination of protein synthesis as demonstrated by *in vitro* minigene experiments. This leads to the retention of a partial intron 5 (80 bp) and the production of a truncated GATA3 protein (p.Ile351Alafs*14).

The comprehensive literature retrieval identified 92 articles that provided valuable insights into HDR syndrome, with a focus on 192 patients (including the patient in our study), who underwent molecular testing for diagnosis (Supplementary Table S2). The patient population was heterogeneous, with Asians accounting for the majority of cases (101/192, 52.60%), including 34 Chinese individuals. Europeans accounted for 50 of the 192 cases (26.04%), whereas Americans and Brazilians accounted for a smaller proportion (8/192, 4.17%). The median age of presentation/diagnosis for HDR was determined to be 5.40 years (range 0–54). It is noteworthy that in recent years, there have been reported cases where a definitive diagnosis was established during the neonatal period (Nakamura et al., 2011; Döneray et al., 2015; Kusakawa et al., 2019). However, the majority of patients still encounter delayed diagnosis. The delayed diagnosis of rare diseases such as HDR syndrome presents a significant challenge, particularly in Asia. In the case of our patient, despite the presence of hearing impairment since birth and the identification of biochemical markers indicative of hypoparathyroidism at 7 months of age, a confirmed diagnosis was not achieved until the patient reached the age of 6 through genetic testing. This delay in diagnosis has resulted in protracted periods of insufficient treatment and rehabilitation, further complicating the timely identification of rare diseases in Chinese primary healthcare institutions. Therefore, the implementation of next-generation sequencing (NGS) technology could serve as a potent tool in facilitating early diagnosis and appropriate management. Advocating for the use of such procedures in Chinese primary healthcare facilities is critical.

HDR syndrome displays highly heterogeneity. In approximately 71.24% (109/153) of patients, the traditional trio of hypoparathyroidism, sensorineural hearing loss, and renal illness was present. Hearing loss stands out as the predominant and prevailing feature of HDR syndrome, reported in approximately 98.90% (179/181) of cases (Table 2). The majority of patients predominantly manifested hypoparathyroidism (167/178, 93.82%), with seizures (56/167, 33.53%) and tetany (29/167, 17.37%) being the most prevalent manifestations. It is interesting to note that 37.13% of patients (62/167) reported no symptoms and only abnormal biochemical indicators. Additional phenotypes associated with hypoparathyroidism included basal ganglia calcification (14/167, 8.38%), intellectual disability (14/167, 8.38%), apnea (3/167, 1.80%), and cataract (3/167, 1.80%). Lack of early management for deafness may be to blame for the intellectual handicap, underlining the importance of early identification and management. Contrarily, renal lesions demonstrated the lowest incidence rate (124/166, 74.70%), which may exhibit age-dependency in its observation, characterized by a mean age of presentation/diagnosis of 13.96 years (range 0–56).

**TABLE 2:** Clinical features of HDR patients.

Clinical feature	Frequency in reported affected individuals with pathogenic *GATA3* variants				
	Missense	Gross deletion	Null	Total	This study
Hypoparathyroidism	36/36 (100%)	22/22 (100%)	122/131 (93.13%)	167/178 (93.82)	+
Seizure	8/36 (22.22%)	10/22 (45.45%)	48/131 (36.64%)	56/167 (33.53%)	+
Tetany	3/36 (8.33%)	4/22 (18.18%)	26/131 (19.85%)	29/167 (17.37%)	+
Basal ganglia calcification	4/36 (11.11%)	2/22 (9.09%)	10/131 (7.63%)	14/167 (8.38%)	+
Apnea	1/36 (2.78%)	1/22 (4.55%)	2/131 (1.53%)	3/167 (1.80%)	+
Cataract	1/36 (2.78%)	2/22 (9.09%)	2/131 (1.53%)	3/167 (1.80%)	-
Mental retardation	4/36 (11.11%)	7/22 (31.82%)	10/131 (7.63%)	14/167 (8.38%)	-
Asymptomatic	17/36 (47.22%)	4/22 (18.18%)	45/131 (34.35%)	62/167 (37.13%)	-
Hearing impairment	38/38 (100%)	25/25 (100%)	141/143 (98.60%)	179/181 (98.90%)	+
Renal lesions	23/35 (65.71%)	19/22 (86.36%)	101/131 (77.10%)	124/166 (74.70%)	-
Renal dysplasia	8/23 (34.78%)	13/19 (68.42%)	52/101 (51.49%)	52/124 (41.93%)	-
Renal agenesis	2/23 (8.70%)	3/19 (15.79%)	28/101 (27.73%)	28/124 (22.58%)	-
Vesicoureteral reflux	5/23 (21.74)	2/19 (10.53%)	23/101 (22.77%)	23/124 (18.55%)	-

Among these reported patients, a total of 103 distinct pathogenetic or likely pathogenetic variants in the *GATA3* gene was identified (Supplementary Table S3), including 40 frameshift, 26 missense, 19 gross deletions, 10 nonsense mutations, and 8 splice-site variants. The distribution of these variants within the *GATA3* gene was nonuniform, as depicted in Supplementary Figure S1. Particularly, a substantial proportion of the variants were localized in exonic regions, accounting for 76 out of the 103 identified variants (73.79%). Exons 3 and 4 displayed a high density of variants, while no variants were detected in exon 1. The majority of the variants (86, 83.50%) were observed only once or twice, and merely 12 single nucleotide variants (12/84, 14.29%) were identified in more than 2 distinct families, indicating a considerable genetic heterogeneity among HDR patients. The incidence rate of novel mutations approximates 41.18% (42/102), which is consistent with earlier literature reports (Upadhyay et al., 2013).

In this study, frameshift, splice-site, and/or nonsense *GATA3* variants were classified as null alleles. Based on their genotypes, the patients were divided into three different groups: (i) missense (*n* = 42); (ii) null (*n* = 125), and (iii) gross deletion (*n* = 25). Most phenotypes were more common in patients with null or gross deletion variations than in those with missense mutations. In the group with missense variants, the prevalence of asymptomatic hypoparathyroidism was noticeably greater (*p* < 0.001). But in contrast to earlier research (Lemos and Thakker, 2020), our study revealed no significant disparity in the age of symptom onset between patients with missense variants and those with null variants (Supplementary Figure S2). A notable differentiation in the age of onset was observed when comparing patients with missense mutations to those with gross deletions (*p* = 0.0157). Similarly, a distinct discrepancy in the age of hearing loss onset was identified exclusively between patients with missense mutations and those with null or gross deletions (*p* = 0.0224 and 0.0145, respectively). Nevertheless, the effect of various mutation types on the age of onset does not appear to be particularly obvious in relation to hypoparathyroidism or renal abnormalities. Furthermore, upon excluding missense mutations, it becomes evident that loss-of-function (LOF) variants affecting distinct structural domains do not exert an influence on the age of symptom onset. The following elements may be responsible for this intriguing outcome: (i) The onset age of several months has been categorized as 0 in prior literature, potentially resulting in imprecise analysis outcomes (Lemos and Thakker, 2020). (ii) Besides hearing loss, the detection of other clinical symptoms in individuals with HDR may necessitate imaging or biochemical assessments. The lack of suitable investigations in some patients and the potential for phenotypic alterations over time contribute to phenotypic variability. Without appropriate examinations, specific characteristics may remain unnoticed and undetected if not evaluated at the appropriate age, resulting in considerable uncertainty. (iii) There are unstudied areas of the *GATA3* gene that could potentially have a significant impact in addition to the ZnF1 and ZnF2 domains. Another possibility is that these variants are located close to critical areas of the protein’s three-dimensional structure. Therefore, clinicians must exercise caution when assessing the genotype-phenotype correlation in HDR syndrome. Similar situations have occurred in our case, despite the variant’s intronic position in the fifth intron, which is thought to have no effect on the functional domains of ZnF1 and ZnF2. However, the affected girl manifested clinical manifestations shortly after birth and experienced recurrent seizures commencing at the age of 7 months. This notable observation further underscores the potential for severe clinical presentations attributed to truncating mutations situated beyond the ZnF1 or ZnF2 regions. Additionally, missense mutations have the power to impair the ability of GATA3 protein to function correctly. Therefore, regular and thorough evaluation of patients’ clinical state is essential for the best possible management and therapy. Moreover, a striking pattern emerged among the 32 observed HDR syndrome pedigrees, wherein 23 pedigrees (71.88%) displayed a phenomenon of genetic anticipation, in which the probands showed symptoms earlier and more severely than their parents carrying the disease-causing mutation. This remarkable discovery emphasizes the progressive nature of the syndrome and accentuates the importance of carefully assessing the severity of symptoms across successive generations.

In this study, we identified a splice site variant, c.1050 + 2T>C, which disrupts the mRNA splicing of *GATA3*, leading to the production of a truncated GATA3 protein (p.Ile351Alafs*14). These groundbreaking findings provide valuable insights into the genetic variations, particularly splicing site variants, that underlie the development of HDR syndrome. In addition, the thorough review part covers all known HDR variants, assisting in the investigation of genotype-phenotype correlations in HDR patients and addressing the complicated issues related to delayed diagnosis and the scarcity of clinical expertise in rare disorders. The progress made in high-throughput sequencing technologies assumes a critical role in unraveling the mysteries surrounding unexplained diseases, and its application is vital in the early diagnosis of HDR syndrome.

## Data Availability

The datasets for this article are not publicly available due to concerns regarding participant/patient anonymity. Requests to access the datasets should be directed to the corresponding authors.
