# Supplementary material for: A GATA3 gene mutation that causes incorrect splicing and HDR syndrome: a case study and literature review
Source: Front Genet. 2023 Aug 25;14:1254556. doi: 10.3389/fgene.2023.1254556 (PMC10485837; doi:10.3389/fgene.2023.1254556)

Supplemental Figure 2. Mean age of diagnosis for each HDR defect based on mutation type. (a) Diagnosis of HDR defects occurred at a earlier age in the 192 patients with missense mutations (n = 42), compared to patients with null mutations (which include frameshift, splice-site, and/or nonsense mutations, n = 125), as well as those with gross deletions (del, n = 25) of the *GATA3* gene. (b) Among the 144 patients with null or gross deletion mutations, they were further categorized into four groups: ZnF1+ZnF2 (Z1+Z2, n = 105), ZnF2 (Z2, n = 16), and others (n = 23). The data were analyzed based on different mutation types (c) and affected regions (d), considering the reported age of diagnosis (when available) for hypoparathyroidism (H), deafness (D), and renal defects (R). Analysis performed with R version 4.2.1. Differences among the groups were assessed using Kruskal-Wallis analysis of variation, followed by Dunn's multiple comparisons test (*p < 0.05, **p < 0.01, ***p < 0.001, ****p < 0.0001; ns, not significant).

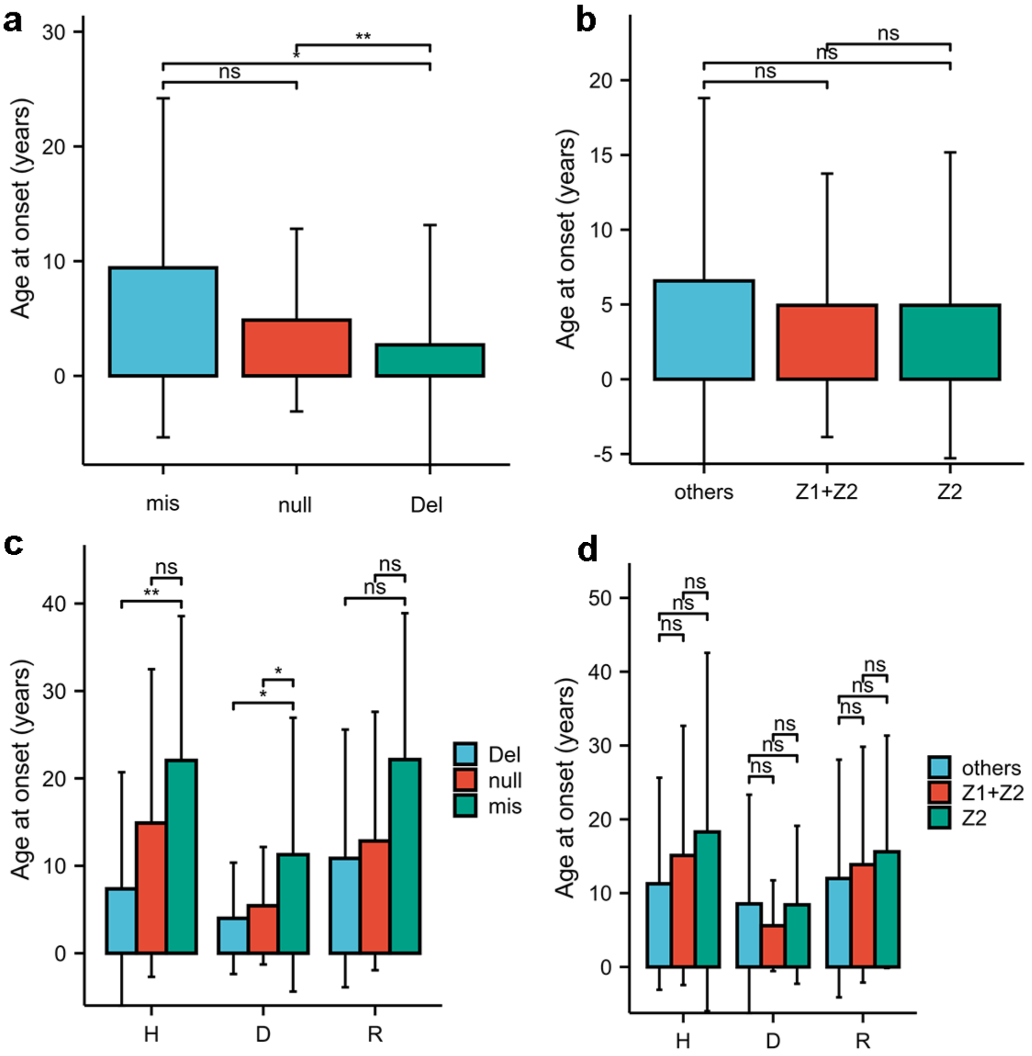

Supplement: Supplementary file 1 [file DataSheet2.PDF]
