# Supplementary material for: A GATA3 gene mutation that causes incorrect splicing and HDR syndrome: a case study and literature review
Source: Front Genet. 2023 Aug 25;14:1254556. doi: 10.3389/fgene.2023.1254556 (PMC10485837; doi:10.3389/fgene.2023.1254556)
Supplement: Supplementary file 3 [file Table3.doc]

**Supplemental Table 3.** Primers used for the amplification of the entire exon 5 and surrounding intron 5 sequence of the wild-type and mutant *GATA3* gene from genomic DNA (*GATA3*-F and *GATA3*-R), and the pcMINI-C-specific primers used for RT-PCR to detect the alterations in splicing by *GATA3* c.1050+2T>C variant in minigene splicing assay (pcMINI-C-F and pcMINI-C-R).

| Primer name | Sequence (5’-3’) |
| --- | --- |
| *GATA3*-F | ACAAGCTTCACAATgCaagtggactgggatc |
| *GATA3*-R | ACAAGCTTCACAATgCaagtggactgggatc |
| pcMINI-C-F | ACTTAAGCTTatgagtgggctttggggtggccggtt |
| pcMINI-C-R | TAGAAGGCACAGTCGAGG |
