# Supplementary material for: A GATA3 gene mutation that causes incorrect splicing and HDR syndrome: a case study and literature review
Source: Front Genet. 2023 Aug 25;14:1254556. doi: 10.3389/fgene.2023.1254556 (PMC10485837; doi:10.3389/fgene.2023.1254556)

Supplemental Figure 1. Schematic representation of *GATA3* variants. The intron-exon structure of the *GATA3* gene is shown. The ZnF1 and ZnF2 indicate zinc finger domains. And the TA1 and TA2 represent transactivation domain. Eighty-four previously reported variants are shown. The variant found in the current study is indicated by a red arrow in intron 5.

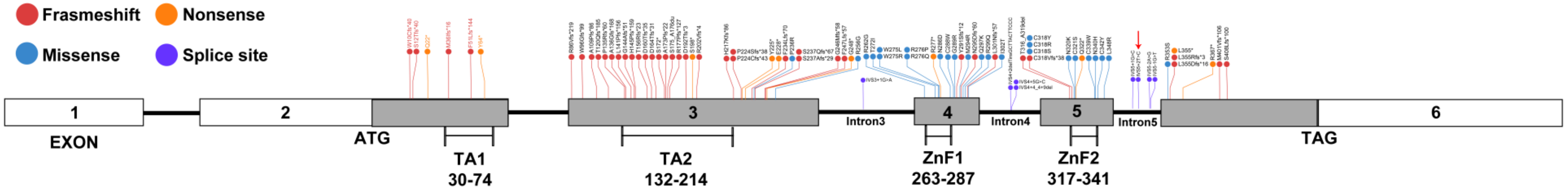

Supplement: Supplementary file 4 [file DataSheet1.PDF]
